# Short-term results (1 year) of vented versus solid polyetheretherketone anchors in treatment of rotator cuff tears

**DOI:** 10.1051/sicotj/2019026

**Published:** 2019-09-04

**Authors:** Marco C. Sarmento, António E. Cartucho, Jacinto M. Monteiro

**Affiliations:** 1 Department of Orthopedic Surgery, Hospital de Santa Maria-Centro Hospitalar Lisboa Norte 1649-035 Lisboa Portugal; 2 Department of Orthopedic Surgery, Hospital CUF Descobertas 1998-018 Lisboa Portugal

**Keywords:** Rotator cuff tear, Polyetheretherketone anchors, Healing process, Solid anchors, Vented anchors

## Abstract

*Background*: Due to the rotator cuff retear after being surgically repaired, some strategies have been developed. The authors verified that the possibility of polyetheretherketone (PEEK) vented anchors promoted a better clinical and healing process than PEEK solid anchors.

*Methods*: A prospective and randomized study was designed with 38 patients treated with PEEK anchors, 18 of whom with vented anchors and 20 with solid ones. Demographic, clinical and radiologic data were collected before and during surgery (time 0) and at 12 months of follow-up.

*Results*: In the final follow-up (12 months), there was no difference in the visual analogic scale (VAS) scale between groups (1.7 points vs 1.9 points; *p* = 0.731), neither in the DASH score (34.2 points vs 23.9 points; *p* = 0.268), nor in absolute Constant score (76.9 points vs 77.3 points; *p* = 0.910). In MRI, 10 patients had their cuff tear healed in the vented group and 15 in the solid group (*p* = 0.173).

*Conclusion*: The new designed vented anchors do not add any advantage when compared to solids ones, at least within the first year after surgery.

Rotator cuff tears (RCTs) represent the most frequent form of complaints of the shoulder girdle which require surgical procedures. They are an important source of pain and shoulder motion restraint, mostly in the overhead activities [1].

The prevalence in population above the age of 65 could achieve up to 40% according to the literature, but the epidemiology is not completely understood as the clinical picture is quite variable, from asymptomatic to continuous pain and a limited range of motion. Thus the diagnosis could be done through several methods with different specificity and sensitivity [2].

The initial treatment approach to degenerative RCT is usually conservative and includes oral medication, physiotherapy, mesotherapy, acupuncture, and local corticosteroid infiltration. However, when these treatments fail, surgical treatment should be an option, yet the reported satisfactory outcome could be as low as 38% [3].

Several prognostic factors have been associated with poor clinical results such as age, symptom duration as well as the quality of tendons, which depend on tear size, muscle atrophy, and fatty infiltration [4].

The failure of the healing process between the tendon and bone interface may be explained by the decreased vascularization within the critical zone, which is poorer in the RCT when compared to healthier tendons [5–7]. Different surgical approaches, surgical devices, and surgical augmentations have been tried in order to boost and improve the healing process of the tendons, increase longevity of clinical results, not to mention making these results last longer. Arthroscopic vs open surgery; single row vs double row vs suture bridge configuration; metal-titanium vs multiple biocomposite material vs polyetheretherketone (PEEK) vs all-suture anchors; acromioplasty vs platelet-rich plasma vs stem cells vs collagen patches – all were used, but without clear, verifiable improvement [1].

The purpose of the present study with vented anchors was to increase the healing potential of the tendons in the enthesis area considering that theoretically the grooved core should promote the migration of stem cells from the cancellous bone of the humeral head to the footprint area, and increase the healing process of tendon repair. This research and comparison between vented and solid anchors has never been compared to humans nor published.

The hypothesis of this prospective clinical study is to verify whether RCTs repaired by vented anchors are clinically and radiologically superior than solid anchors.

## Material and methods

This prospective, randomized, clinical study assessed the use of two different PEEK anchors, vented and solid. The architecture of the vented anchor, more grooved, when compared to the solid one, hypothetically allows a more efficient harvest of stem cells and inflammatory cells to the footprint area, stimulating the healing of the torn anchored tendons.

The null hypothesis is that the clinical results after 1 year of follow-up in both groups of patients are the same.

The clinical study was approved by the ethics committee of the hospital.

### Patient data

Two groups of patients with posterosuperior degenerative RCT, diagnosed by MRI were randomly created: in the first group (group 1), 18 patients were treated using vented PEEK anchors; in the second group (group 2), 20 patients were treated using solid PEEK anchors. Both groups were treated with the arthroscopic approach by 1 of the 2 senior surgeons (MS and AC).

### Inclusion and exclusion criteria

Patients were included granted that (1) they had a posterosuperior degenerative, chronic, and complete detachment of at least the supraspinatus with intact subscapularis or with a tear which did not require any surgical procedure; (2) they were aged between 45 and 80; (3) the patient had a previous MRI with a diagnosis of complete RCT; (4) there was a complete repair with a perfect coverage of the footprint, if necessary with enlarged release of the cuff retraction. In all patients, a tenotomy of the long head of the biceps was performed, regardless of its delamination, tenosynovitis, inflammation, subluxation, or dislocation.

Exclusion criteria were: (1) massive irreparable RCT, (2) when the repaired tear was only partial without full coverage of the footprint, (3) in partial, bursal, or articular, cuff tears, (4) when any prior surgery in the shoulder, whatever the reason, or any reoperation in the same shoulder after the inclusion in the study was performed, (4) any history of fracture of the scapular zone (clavicle, proximal humerus or scapula), (5) subscapularis tear, isolated or in combination, which required surgical reinsertion and (6) any kind of workers’ compensation claims or profit involved.

Contraindication for surgery was severe muscle atrophy or fatty infiltration according to the classification of Goutallier et al. [8] adapted by Fuchs et al. [9] for MRI.

The patients were included in the study protocol only after the diagnosis was verified and confirmed at the beginning of the surgery.

### Randomization

Randomization was performed according to the CONSORT rules [10] in two different blocks, one with 18 patients and the other with 20 patients.

In the test group (group 1), we used a vented PEEK anchor in the insertion of the tendons in the footprint. In the control group (group 2), we performed the same type of arthroscopic reparability but using a solid PEEK anchor.

After the informed consent for the surgery and for the study, all of the patients were blinded for the type of anchor/group they were allocated to.

All clinical assessments, scores, and radiographic evaluations were executed by the same observer (MS).

### Surgical technique and postoperative care

All patients were operated in beach chair position, with a combined general anesthesia and brachial plexus block. In the articular zone, a general inspection was performed; thus all the anatomic structures were identified. When the diagnosis of RCT was established, and the patient was elected for the protocol, an articular synovectomy and tenotomy of the long head of biceps were performed. Passing to the subacromial space, and after removing the bursa and the edges of tendons regularized, the dimension, the shape, and the retraction of the tear were measured and registered. Hereafter, the soft tissue and cortical bone in the upper surface of the great tuberosity, in the footprint area of the cuff, were removed and abraded with a shaver and a hole field with less than 3 mm in depth was created with a punch to promote a bloody layer to increase the healing process of the cuff. A single- or double-row technique was applied depending on the geometry, dimension, and mobilization of the tendons after the insertion of the medial row: in small tears, less than 1 cm in antero-posterior and latero-medial direction, a single row of double-loaded PEEK anchors was used; in medium-to-large tears, more than 1 cm in antero-posterior or latero-medial direction, and when the footprint was not completely covered by the medial row, a lateral transosseous equivalent row magnified the tendon-bone contact. There was no difference between the two groups in the surgical approach and steps during the surgery apart from the type of medial anchor: vented bioabsorvable PEEK in group 1 and solid bioabsorvable PEEK in group 2.

A subacromial decompression with acromioplasty was not performed to avoid creating bias which could influence the results of the healing process attributed to stem cells derived from the acromion process.

Shoulders were immobilized for a 6-week period after surgery, using a sling in a slight, abduction position all day, except for bathing, and getting dressed or undressed and during the passive exercises or physiotherapy. Patients were instructed to mobilize the elbow, wrist, and hand immediately after as well as when also doing their passive exercises (pendular exercises). After removing the surgical stitches, 1 week after the surgery, they were sent to a physiotherapist to start the passive motion exercises under supervision until the fourth week. Then, active-assisted range of motion was introduced as the next recovery step that normally occurs in the sixth week, when the sling was progressively removed, and the active motion exercises were encouraged if full range of motion passively was obtained.

Strengthening exercises were introduced between the 12th and 16th weeks, depending on the evolution of the range of motion and pain control. Returning to work and sport activities were dependent on individual characteristics and performance.

### Clinical assessment

#### Evaluation

Patients were assessed during the week before the surgery for baseline and then at 12 months for final follow-up.

### Clinical outcome measures

Three different clinical outcomes were applied preoperatively and postoperatively:

1. The visual analogic scale (VAS) to evaluate pain perception before surgery and a year later for a final clinical evaluation [11–13];
2. DASH score for the double purpose of patient self-evaluation of symptoms and ability to perform activities, in a Portuguese translation, before surgery and at final follow-up [14];
3. The absolute Constant and Murley Score in the same periods [15–17].

### Radiographic outcome measures

All patients were evaluated pre-operatively and 12 months after surgery by MRI. The extent of RCT on the pre-operatively scan was confirmed during the arthroscopic procedure by direct visualization. With the scope in the posterior and lateral portal, the location, size, retraction, and morphology of the tear were registered. The size and location of the tear was classified according to Cofield [18]. Retraction in the frontal plane, in the cut where the largest latero-medial dimension was measured, was done using Patte’s [19] classification.

At the final follow-up MRI (12 months), the integrity of the cuff repair was verified. The five grade Sugaya et al.’s [20] classification was used and the healing process was subdivided into two major groups: 1 – cuff repaired (grade I–III), and 2 – cuff not repaired (grade IV–V).

The scans were all accessed by the same senior surgeon (MS).

### Statistics

Statistical analyses were performed using SPSS 24.0 software (IBM Corp, Armonk, NY, USA). Data are presented as mean ± standard deviation of the mean. The mean values were compared using the Mann–Whitney test for unpaired groups and the Wilcoxon test for paired groups for continuous variables and the χ^2^ test or the Fisher exact test for categoric variables. The Spearman correlation coefficient was used to test the quantitative relationships between variables. A difference of *p* < 0.05 was statistically significant. We determined the study sample size with a power analysis to provide statistical power (80%) at a level of 0.05.

## Results

### Baseline demographics

Thirty-eight patients (26 women and 12 men) met the inclusion criteria and were randomized between two similar groups, 18 in group 1 and 20 in group 2. The average age was 64.3 (range of 48–75 years of age) for total patients, with an average age of 65.3 for group 1 (range 51–75 years of age) and 63.4 for group 2 (range 48–73 years of age). The average duration of symptoms before surgery was 9.7 months for group 1 and 14.6 months for group 2. There were no smoker patients in both groups. There were no significant differences in baseline characteristics between the two groups.

Before surgery, seven patients in group 1 and five patients in group 2 were infiltrated in the subacromial space at least once, with a maximum of three infiltrations of methylprednisolone. All patients in both groups were first treated conservatively before surgery through oral medication (NSAID and/or analgesic), physiotherapy and mesotherapy, or both.

In group 1, retraction on the supraspinatus tendon in frontal plane (MRI) was classified as less or equal to 2 in all patients except for one, as a result muscle atrophy was less or equal to 2 in all patients apart from one; fatty infiltration was less or equal to one in all except for two patients; similarly, in group 2, tendon retraction was less or equal to two except for two patients, muscle atrophy was less or equal to two in all patients, and fatty infiltration was less than one in all patients. The Mann–Whitney *U* test considered the two groups similar for these variables.

The real dimension of the tear measured intra operatively was 2.13 cm in anteroposterior plane and 2.15 cm in the lateromedial plane, in group 1, and 1.76 cm and 1.54 cm in group 2, respectively (*p* > 0.05) (Table 1).

**Table 1:** Comparison of pre-operative epidemiological and clinical parameters.

Parameter	Group 1 (*n* = 18)	Group 2 (*n* = 20)	*p* value*
Gender distribution			.251
Female, No	14	12	
Male, No	4	8	
Age at surgery, mean (range) years	65.3 (51–75)	63.4 (48–73)	.395
Average symptoms before surgery, mean (range) months	9.7 (3–24)	14.6 (1–24)	.263
Smokers	0	0	
Physiotherapy	8	10	.740
Oral medication	15	20	.066
Subacromial infiltration	7	5	.308
RMN retraction (supraspinatus)α			.128
None	1	5	
1	5	6	
2	11	7	
3	1	2	
RMN atrophy (supraspinatus)β			.131
None	5	9	
1	8	9	
2	4	2	
3	1		
RMN fatty infiltration (supraspinatus)β			.063
None	10	15	
1	6	5	
2	2		
Rupture dimension (mm)			
Antero-posterior, mean ± *SD*	2.13 ± 0.25	2.53 ± 0.25	.218
Latero-medial, mean ± *SD*	2.16 ± 0.92	1.76 ± 0.88	.184

Group 1: vented anchors; Group 2: solid anchors; *SD*: standard deviation.

* According to the Mann–Whitney test (*p* < .05 indicates statistical significance).

α According to Patte [19].

β According to Goutallier et al. [8] modified by Fuchs et al. [9].

**Figure 1:**
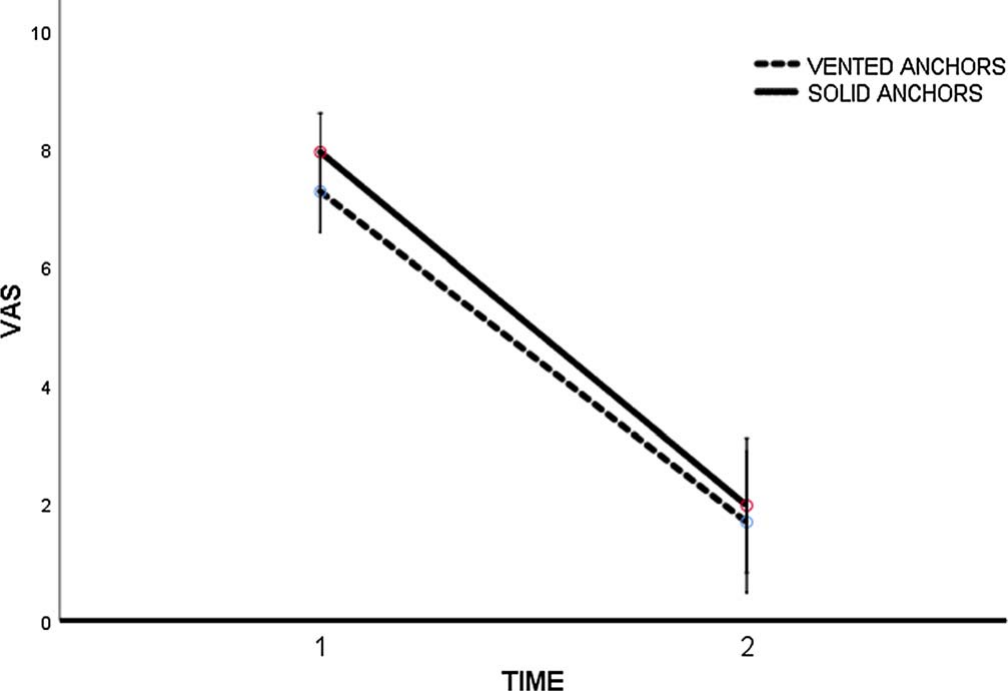
VAS evolution. Time 1: pre-op; Time 2: 12 months.

The initial VAS score was 7.3 points (*SD* ± 0.3), in group 1, and 7.9 points (*SD* ± 0.3) in group 2. At the final follow-up (12 months) the results were 1.7 points (*SD* ± 1.98), in group 1 and 1.9 points (*SD* ± 0.6) (*p* = 0.731) in group 2 (Figure 1).

**Figure 2:**
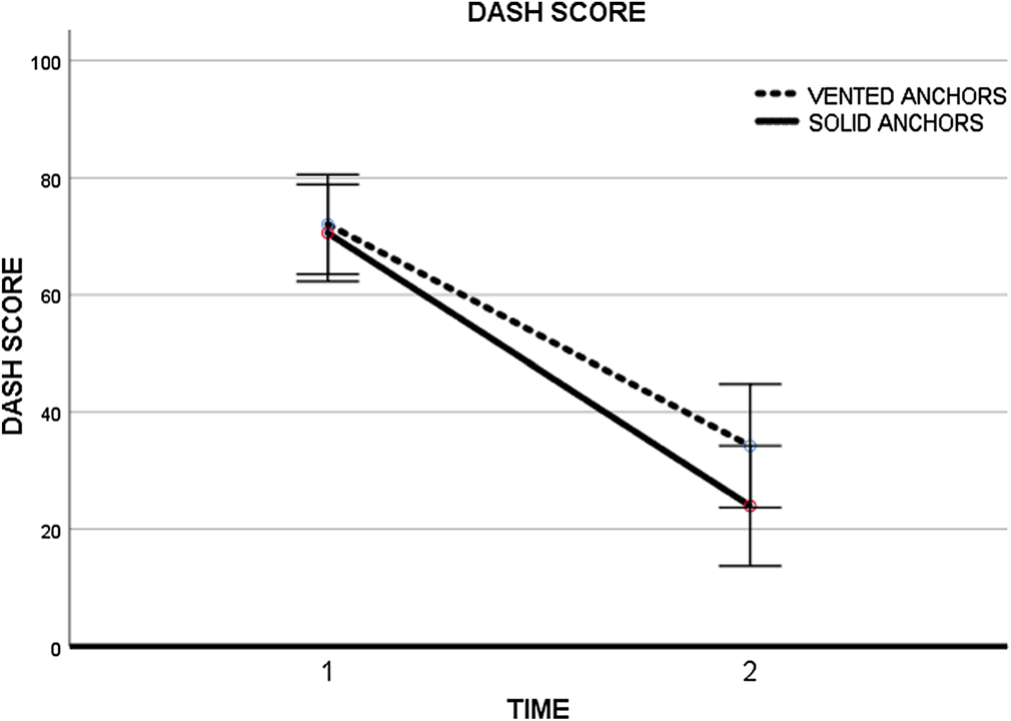
DASH Score evolution. Time 1: pre-op; Time 2: 12 months.

In DASH score, the starting point for group 1 was 72.1 points (*SD* ± 4.1) and 70.6 points (*SD* ± 4.1) for group 2. The first group decreased its values to 34.2 points (*SD* ± 5.2) at the final follow-up and the second group had got 23.9 points (*SD* ± 5.1) (*p* = 0.268) (Figure 2).

**Figure 3:**
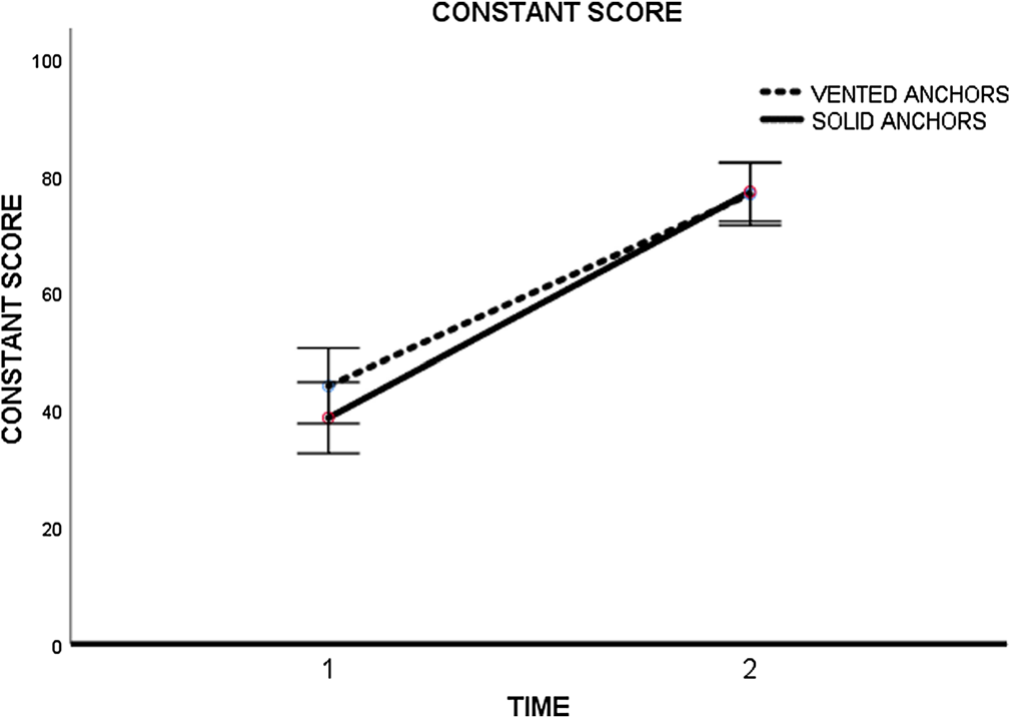
Constant Score (absolute value in points) evolution. Time 1: pre-op; Time 2: 12 months.

The pre-surgical evaluation of Constant score gave 44.1 points (*SD* ± 3.2) and 38.7 points (*SD* ± 3.0) to groups 1 and 2, respectively. In the final evaluations, the score was 76.9 points (*SD* ± 2.6) for group 1 and 77.3 points (*SD* ± 2.5) for group 2 (*p* = 0.910) (Figure 3) (Table 2).

**Table 2:** Comparison of clinical parameters pre-operatively and 12 months post-operatively in both groups and between groups.

Parameter	Group 1			Group 2			
	Pre-op	Post-op at 12 months	*p* value	Pre-op	Post-op at 12 months	*p* value	*p* value**
VAS score, mean ± *SD*	7.3 ± 0.3	1.7 ± 1.98	.001	7.9 ± 0.3	1.9 ± 0.6	.001	.731
DASH score, mean ± *SD*	72.1 ± 4.1	34.2 ±5.2	.001	70.6 ±4.1	23.9 ± 5.1	.001	.268
Constant Score, mean ± *SD*	44.1 ± 3.2	76.9 ± 2.6	.001	38.7 ± 3.0	77.3 ± 2.5	.001	.910

Group 1: vented anchors; Group 2: solid anchors; *SD*: standard deviation.

* According to Wilcoxon test (*p* < .05 indicates statistical significance).

** *p* value of final results between Group 1 and Group 2.

All patients (38), 18 in group 1 and 20 patients in group 2, had an MRI at the final endpoint of 12 months. Dividing Sugaya et al.’s classification [20] in two groups: the first, in which there was healing of the tear, total, or partial (type I–III) and in the second one, in which there was not that capability, with maintenance or enlargement of the tear (type IV and V), we verified that 10 patients had healed and eight hadn’t healed the tendons in group 1, compared to 15 patients who healed and five who did not heal the tendons in group 2 (*p* = 0.173) (Table 3).

**Table 3:** Sugaya’s classification at 12 months follow-up.

Group 1		Group 2	
Healed (Sugaya I–III)	Not healed (Sugaya IV–V)	Healed (Sugaya I–III)	Not healed (Sugaya IV–V)
10	8	15	5

Group 1: vented anchors; Group 2: solid anchors; *p* = 0.173.

* According to Wilcoxon test (*p* < .05 indicates statistical significance).

## Discussion

A large amount of research has been done to promote the tendon healing process in RCTs, focusing on the quality of the tendon [1, 21] and vascularization [22–24], which are two of the most important factors.

The quality of the tendon could not be changed in the surgical moment, but its blood supply should be improved, stimulating pluripotent stem cells from the subacromial bursa or bone marrow, which, after migration and differentiation, are capable of enhancing the cuff tear healing process [25].

Taking into account the animal research done by Kida et al. [26] and Caplan [27], the authors’ study proposal was to bring to the human field the usefulness of vented anchors in this pathology, whose characteristics were described.

This is the first time the two types of anchors were compared to humans, as far as we know.

The two groups in comparison were demographically similar in terms of age, gender, distribution, and time of symptoms, and independent variables were considered in the final results. The same was verified to MRI analysis before surgery, related to the dimension of the tear and quality of remaining tendons and confirmed during the arthroscopy by measuring the tear in antero-posterior and latero-medial plane.

Statistically, there were no differences in the starting point between groups when the clinical evaluation was done by the three clinical parameters: VAS, Constant score, and DASH score.

All patients clearly improved their clinical scores at final follow-up, a year after surgery.

As in the initial clinical evaluation, the final results of the VAS, Constant score, and DASH score were equal for both groups, without any statistical difference between them.

The indirect healing measure of cuff tendons analyzed by MRI showed no difference between groups, with a healing rate of 10 patients out of 18 in the vented group and the rate of 15 out of 20 in the solid group.

This study has some limitations that could be considered as weak points. Two surgeons were enlisted in the surgical treatment of the patients, which despite the same medical school philosophy and surgical steps, could be considered a performance bias. Secondly, 1-year follow-up time could be short in clinical terms because the results after this period are expected to worsen progressively even though the retear rate happens customarily in the first 6 months when MRI is analyzed. The error type II is a statistical error which is related to the dimension of the sample. However, with a greater dimension of the sample, the conclusions could be more considerable.

In this clinical study, the results are equal when comparing patients in whom solid or vented PEEK anchors were used to suture RCT in similar demographic patients and surgical method. The substrate that was based in the research, in which vented anchors added value promoting the collection/harvest and migration of stem cells by the increasing blood supply to the footprint area thus increasing the healing process, was not proven.

## Conclusion

The expectation conceived that the use of the vented anchors increasing the healing process in the surgical treatment of RCTs based on animal research was not confirmed in the human field.

In this clinical study, two different groups with similar clinical and radiological parameters at the starting point achieved the same results at the 1-year follow-up.

According to our early results, vented anchors don’t add value in terms of healing process and clinical results compared to standard solids ones.
